# The invasion of fall armyworm and green pest control: an analysis of farmers’ willingness to adopt genetically modified insect-resistant maize in Southwest China

**DOI:** 10.1080/21645698.2025.2497909

**Published:** 2025-05-27

**Authors:** Yanfang Huang, Minglong Zhang, Xiangping Jia, Fengying Nie, Xianming Yang, Kongming Wu

**Affiliations:** a Institute of Plant Protection, Chinese Academy of Agricultural Sciences, Beijing, China; b Agricultural Information Institute, Chinese Academy of Agricultural Sciences, Beijing, China

**Keywords:** Adoption willingness, fall armyworm, genetically modified insect-resistant maize, genetically modified technology, green pest control

## Abstract

The invasion of fall armyworm poses a serious threat to global food security, necessitating the urgent promotion of environmentally friendly pest control technologies. As a key strategy in green pest control, the effectiveness of genetically modified (GM) insect-resistant crops largely depends on the extent of farmer acceptance. Based on survey data from 426 GM maize pilot farmers in Yunnan Province, this study employs an ordered Logit model to analyze the key factors influencing farmers’ adoption intentions from a behavioral decision-making perspective. The results indicate that (1) cognition, value orientation, and social trust are the primary drivers of farmers’ willingness to adopt GM maize; (2) the impact of risk perception is context-dependent, exerting a significant positive moderating effect on planting decisions among farmers with higher levels of social trust; (3) planting experience reinforces the effects of cognition and social trust and amplifies the role of value orientation. This study provides policy-relevant insights for the industrial promotion of GM crops and the green control of fall armyworms, with important implications for safeguarding national food security.

## 1. Introduction

The invasion of fall armyworm (*Spodoptera frugiperda*) poses a severe threat to global food security. As a long-distance migratory pest, fall armyworm is native to tropical and subtropical regions of the Americas and has spread to over 70 countries across Africa, Asia, and Oceania since 2016,^1,2^ causing significant damage to crops such as maize.^3^ Statistics show that in African countries, fall armyworm leads to an annual maize yield loss of 8.3 to 20.6 million tons,^4^ while in parts of Asia, it has affected 2 million hectares of maize, resulting in yield reductions exceeding 10%.^3^ Since its invasion of Yunnan Province, China, in January 2019, fall armyworm has rapidly spread northward, reaching 27 provinces, municipalities, and autonomous regions, with an affected area of nearly 1.2 million hectares.^5^ It has become a major pest of maize production,^6^ with potential annual economic losses estimated at $5.4 billion to $47 billion,^7^ posing a severe threat to China’s food security. In summary, effective control of fall armyworm is critical to ensuring maize production and safeguarding food security.

Chemical control remains the primary short-term measure to combat fall armyworm. Due to their rapid and effective action, chemical pesticides continue to be the main method for field control of fall armyworm.^5,8^ Among them, organophosphates and carbamates, as traditional insecticides, were the first chemicals used to manage fall armyworm.^9^ In recent years, insecticides such as *Spinetoram*, *Emamectin Benzoate*, and *Chlorantraniliprole*, which show significant efficacy against fall armyworm larvae and eggs,^10^ have been widely adopted for its control.^11^ However, long-term reliance on chemical pesticides not only increases pesticide input costs^12^ but also degrades soil quality and disrupts biodiversity,^13,14^ thereby endangering agricultural production, the ecological environment, and human health.^15^ Consequently, as the spread and outbreaks of fall armyworm persist, there is an urgent need to promote the development of green pest control technologies to achieve sustainable management.^16^

Cultivating genetically modified insect-resistant varieties is one of the key strategies for the green control of fall armyworm (*S. frugiperda*). Since 2000, countries have gradually shifted to using BT insect-resistant maize for managing fall armyworm. For example, the insect-resistant maize variety Mp708 developed in the United States delays fall armyworm larval development by damaging the peritrophic membrane of the larvae.^17^ To date, China has successfully developed single-gene Cry1Ab maize, double-gene Cry1Ab + Cry2Aj maize, and double-gene Cry1Ab + Vip3Aa maize lines.^18^ These varieties have demonstrated excellent control efficacy against fall armyworm^19^ and represent a green pest control technology that could gradually replace chemical control.^20^

Meanwhile, GM crops have become a topic of debate among consumers, farmers, scientists, non-governmental organizations, and the biotechnology industry.^21^ Among these stakeholders, farmers are the primary suppliers of GM crops, and their willingness to pay for such crops directly affects the promotion of GM insect-resistant varieties and the effectiveness of fall armyworm control. In fact, addressing the issue of farmers’ willingness to cultivate GM crops is essential to transforming the demand for GM insect-resistant varieties into a practical market reality.^22^ Therefore, farmers’ attitudes toward planting GM insect-resistant varieties are crucial for the commercialization of GM crops. Understanding farmers’ acceptance and willingness to pay is essential for assessing the role of GM technology in safeguarding national agricultural production and food security.

Currently, differences in the perception and acceptance of genetically modified crops across countries add complexity to national decision-making processes.^23^ In recent years, researchers have primarily analyzed farmers’ acceptance and willingness to pay for GM crops from the following perspectives: the influence of farmers’ own endowment characteristics, such as age and education level, on planting behavior;^24^ the impact of farmers’ perceptions of GM crops on their acceptance levels;^25,26^ and the factors influencing farmers’ perceptions of GM crops.^27–29^ However, research on Chinese farmers’ perceptions of GM crops and their influence on adoption willingness remains insufficient. Some studies have focused on farmers who have not actually planted GM crops, leading to biased results in willingness-to-pay estimates.^30–32^ Other studies have rarely included farmers’ perceptions of environmental risks as factors influencing technology adoption.^22,33^ Additionally, existing studies often demonstrate a relatively shallow understanding of GM technology.^34^ Even when addressing deeper cognitive levels, they generally explore only the correlations between variables without examining their underlying mechanisms.^35^ Specifically, there is a notable lack of research on the interaction mechanisms between farmers’ perceptions of GM technology, social trust, and willingness to adopt GM crops.^22^

This study focuses on maize farmers in Pu’er City, key pilot areas for genetically modified crops in China, to analyze the direct effects of cognitive level, risk perception, and value orientation on farmers’ adoption of GM technology. Furthermore, it explores whether social trust can enhance the positive impact of these factors on the adoption of GM technology. This research not only provides a novel perspective for addressing the issue of low technology adoption rates caused by insufficient farmer awareness but also holds practical significance for the development of green prevention and control technologies against the fall armyworm. By offering insights into revitalizing China’s maize industry from an alternative angle, this study contributes to addressing global food security challenges.

## 2. Theoretical Framework and Research Hypotheses

### 2.1. Theoretical Farmwork

According to the rational agent hypothesis, farmers’ production decisions are guided by the principle of profit maximization.^36^ This implies that farmers are likely to adopt genetically modified crops if they reduce input costs or increase yields.^37^ However, a purely economic perspective often overlooks the complex psychological factors influencing decision-making. In reality, human rationality lies between full rationality and irrationality, and decision-making is susceptible to perceptual biases during problem identification and resolution.^38^ Behavioral Decision Theory (BDT) offers a novel approach to address the limitations of the full rationality assumption in complex decision-making scenarios. BDT posits that in real-world contexts characterized by high uncertainty and complexity, individuals face constraints in cognitive capacity, information processing, and computational ability, making it challenging to achieve optimal decisions as predicted by full rationality. When applied to agricultural research, farmers’ decisions to adopt GM crops are not solely driven by cost-benefit analyses but are significantly influenced by psychological factors such as cognition, perception, and value orientations,^34^ following a specific “cognition-intention-behavior” decision-making framework.

Traditional BDT, due to its relatively narrow focus, struggles to explain complex decision-making behaviors fully. Consequently, numerous scholars have expanded the theory by incorporating psychological constructs such as attitudes and perceptions. Extensive research has confirmed that multiple factors drive farmers’ technology adoption behaviors. For instance, Yuriev et al.^39^ demonstrated that prior experience, risk perception, and value orientations play critical roles in farmers’ decisions to adopt new agricultural technologies. Additionally, social trust has been identified as a significant determinant of behavioral decisions.^40,41^ From a psychological perspective, diverse value orientations – such as egoism, altruism, and biospheric values^42^—influence individuals’ attitudes and intentions, leading to significant behavioral variations.^43^

**Figure 1.**
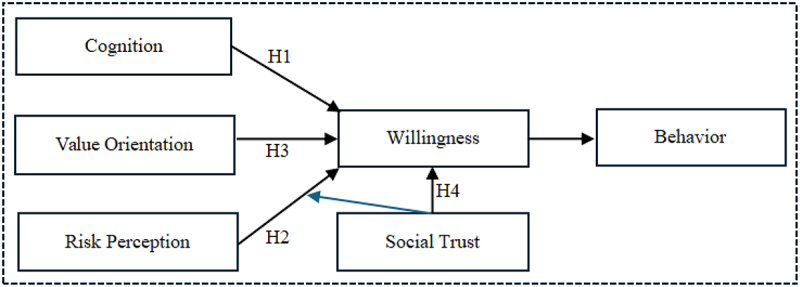
Theoretical analytical framework.

Given these, the study adopts the “cognition-intention-behavior” framework of BDT as its core theoretical basis while incorporating relevant extended constructs from the theory. A series of hypotheses regarding factors influencing farmers’ willingness to adopt insect-resistant GM maize are proposed, and a theoretical analytical framework is constructed accordingly (Figure 1).

### 2.2. Research Hypotheses

Farmers’ cognition about genetically modified technology directly influences their acceptance and willingness to adopt GM crops).^22,44,45^ Farmers often face considerable risks when deciding to adopt new technologies. Consequently, unless they have a thorough understanding of the new technology, most farmers are unlikely to adopt it, highlighting the critical role of cognition in the adoption process.^46–48^ For example, several early studies conducted in Europe found that farmers’ perception that planting GM crops could reduce production costs was a major factor influencing their willingness to adopt such crops.^49–51^ In Argentina, the perceived cost-reduction benefit of herbicide-resistant soybeans was identified as a key determinant of farmers’ willingness to plant them.^52^ Similarly, a study in Pakistan revealed that farmers’ acceptance of GM crops increased as their understanding deepened.^53^ In addition, prior research in China reached the same conclusion, showing that farmers’ cognition of GM rice had a significant positive impact on their willingness to plant GM rice.^32^ Based on this, the following hypothesis is proposed:

H1. The higher the level of knowledge, the greater the farmers’ willingness to adopt insect-resistant GM maize.

Risk perception is a primary factor influencing farmers’ attitudes toward and willingness to adopt genetically modified technology.^54–56^ Generally, two main types of risk perception can be identified: unknown risk and fear risk,^55^ which have been evaluated in existing research. Unknown risk factors increase farmers’ uncertainty and hesitation toward adopting GM technologies. Fear risk factors amplify the perception of GM technology as frightening, dangerous, and potentially harmful to future generations.^57^ For instance, Balzekiene et al.^58^ revealed that people’s perception of unknown risks related to GM technology leads to delays in adopting the technology. Another study found that farmers’ risk perception of GM technology is heightened due to the unknown consequences of its use.^59^ Over time, this can lead to a rejection of GM crop cultivation. For example, farmers perceive GM crops as potentially damaging the soil environment by disrupting biodiversity, increasing ecological risks.^60^ Siegrist et al.^61^ found that farmers’ high-risk perception of GM crops indirectly affects their attitudes toward GM technology. In conclusion, farmers’ risk perception influences their beliefs about and willingness to adopt new technologies. Therefore, the following hypothesis is proposed:

H2. The higher the level of risk perception, the lower the farmers’ willingness to adopt insect-resistant GM maize.

Value orientation plays a critical role in explaining specific beliefs and behaviors and serves as a primary predictor of these outcomes.^62,63^ In environmental psychology and social issue research, numerous studies have analyzed the relationship between values, beliefs, and actions that are significant for environmental and social development.^64,^^65^ Additionally, in social dilemma research, various scholars have explored the important role of value orientation in explaining behavior,^66–68^ noting that individuals with a social value orientation are more focused on optimizing outcomes for others, while those with a self-value orientation are more focused on optimizing outcomes for themselves. Currently, there remains considerable ethical controversy surrounding the cultivation of genetically modified crops,^37^ with some expressing concerns that GM crops could cause damage to ecosystems.^60^ However, the ethical egoism argument posits that all human actions are self-interested,^69^ and previous studies have emphasized that self-efficacy is an important factor influencing the adoption of new technologies.^70–72^ Therefore, the following hypothesis is proposed:

H3. The stronger the self-oriented value orientation, the greater the farmers’ willingness to adopt insect-resistant GM maize.

Social trust is a critical factor influencing farmers’ technology adoption.^41^ Since the 1950s, trust research has developed along three major theoretical paths: the “interpersonal trust” approach in psychology, the “rational calculation” approach in economics, and the “social trust” approach in sociology.^73^ Based on the assumption of the rational economic actor, distrust is considered the default state. Individuals choose to trust only when it is necessary to gain benefits while ensuring their existing interests are not harmed.^74^ In the context of agricultural technology dissemination, the degree of trust significantly influences public acceptance of genetically modified technology.^75^ For instance, Feng et al.^76^ employed regression analysis and found a significant positive correlation between farmers’ trust in expert systems, such as scientists, and their willingness to plant GM crops. Furthermore, due to the significant uncertainty in the GM agricultural product market, severe “market failures” often occur.^32^ Therefore, government intervention and regulation are necessary to promote the industrialization of GM crops actively.^77^ For example, Dai and Wang,^78^ demonstrated that the level of government support significantly impacts farmers’ willingness to cultivate GM crop varieties. Based on the above, the following hypothesis is proposed:

H4. The higher the level of social trust, the greater the farmers’ willingness to adopt insect-resistant GM maize.

## 3. Data and Methods

### 3.1. Data Source and Sample Description

**Figure 2.**
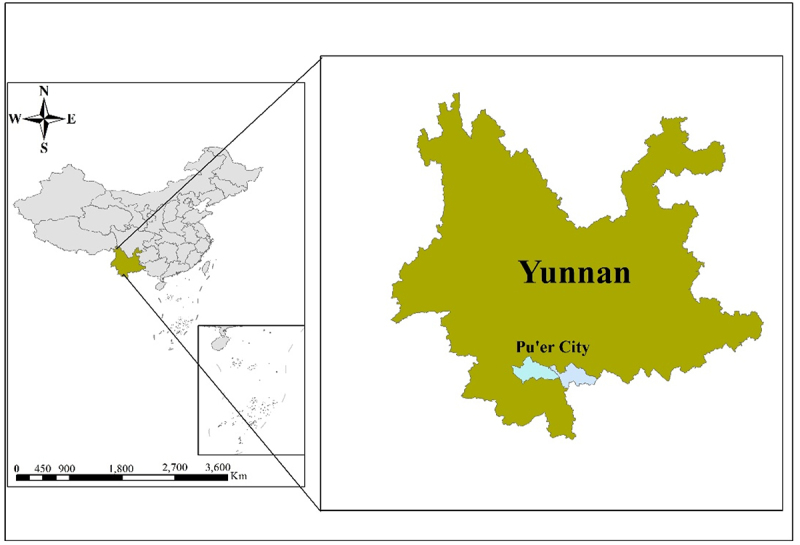
Research area.

This study is based on household survey data collected by the research team in County A and District B of Pu’er City in Yunnan Province, China, in March 2023 (Figure 2). Pu’er city is located in Yunnan Province in southwest China, the region where the fall armyworm was first detected in the country in 2019, posing a severe threat to the local maize industry. Both counties served as pilot areas for promoting insect-resistant GM maize varieties from 2022 to 2023, with an average altitude of 1,300–1,400 meters. Local agricultural production primarily involves growing crops such as maize and rice on small, fragmented plots owned by individual smallholder farmers, who make independent production decisions.^79^

To ensure methodological robustness, this survey employs a tripartite sampling approach combining multistage, stratified, and random techniques to ensure sample representativeness. In the first stage, County A and District B in Yunnan Province were selected as sample sites, considering the status of the 2022 genetically modified maize pilot project, geographical location, and maize farming patterns. In the second stage, 13 sample villages were chosen using stratified sampling based on factors such as the pilot project implementation, geographical location, economic development, and maize planting area. Specifically, three townships were selected from each sample county, and 2–3 villages were randomly selected from each sampled township. In the third stage, household lists provided by village leaders were used to number all households, and 36 households were randomly selected from each sampled village.

The data collection process was conducted in several phases. First, we conducted systematic surveyor training covering questionnaire comprehension, data recording standards, quality control essentials, strategies for handling unexpected situations, and simulated “surveyor vs. farmer” exercises. Second, prior to formal data collection, a pre-survey was conducted with a representative small sample of farmers to evaluate the stability of data collection tools, the rationality of operational procedures, and the reliability of data quality. This allowed for the timely identification and correction of potential issues. Third, face-to-face household interviews were employed during the formal data collection phase. One surveyor communicated with a farmer one-on-one completed the questionnaire on-site and verified critical data in real-time to ensure accuracy and completeness. Additionally, at the end of each day’s data collection, surveyors immediately conducted questionnaire reviews, collectively discussing common issues identified during the process to ensure proper resolution.

The survey questionnaire covered various aspects, including household demographics (e.g., gender, age, education level), agricultural production (e.g., land use, planting, fertilization, irrigation, harvesting, and sales), income and expenditure, risk and preferences, household decision-making power, information sources, planting and consumption intentions, cognition and perception, value orientation, food consumption, health (both mental and physical), as well as training and management related to crop cultivation. The survey was conducted through one-on-one interviews between farmers and trained undergraduate students and teachers. A total of 468 paper questionnaires were distributed. After excluding invalid samples with missing key information or outliers, 426 valid responses were obtained, resulting in an effective response rate of 91.0% (as shown in Table 1).

**Table 1.** Sample size description.

County	Town	Village	Households
County A	3	7	230
District B	3	6	196
Samples (N)	6	13	426

### 3.2. Variable Selection

*Dependent variable*. The dependent variable of this study is farmers’ willingness to adopt genetically modified insect-resistant maize varieties. This was measured by asking farmers whether they were willing to plant GM insect-resistant maize varieties in 2023. Specifically, a five-point Likert scale (1–5) was used: 1 = absolutely unwilling; 2 = relatively unwilling; 3 = neutral; 4 = relatively willing; 5 = very willing. Generally, a five-point Likert scale can more accurately reflect farmers’ true willingness to adopt GM technology compared to a binary variable.^80^

*Key explanatory variables*. This study constructed four key explanatory variables using Principal Component Analysis (PCA) (Table 2). First, drawing on contemporary mainstream research, the GM cognition index includes farmers’ perceptions of GM crop quality (e.g., kernel plumpness, absence of mold), perceptions of GM crop yields, and perceptions of GM crop inputs (e.g., pesticide and labor inputs).^22,81^ Additionally, the index incorporates farmers’ perceptions of the relationship between consuming GM foods and human health, feeding GM feed to livestock and livestock health, and planting GM crops and biodiversity. Second, the GM perception index encompasses farmers’ risk expectations across five dimensions: climate change, pest outbreaks, soil conditions, ecological environment, and the maize market. Third, the value orientation index is based on social value research^82^ and is measured by asking farmers whether they prioritize self-interest (egoistic), others’ interests (altruistic), national development strategies (pro-national), or environmental protection (pro-ecological) when deciding to plant GM crops. Finally, the social trust index is constructed based on the diffusion of innovation theory and social network theory commonly used in agricultural technology adoption studies.^40,83^ It is measured by asking farmers whether they are willing to try GM insect-resistant maize varieties recommended by government officials, relatives, neighbors, village cadres, agricultural extension agents, or seed companies.

**Table 2.** Variable measurement and indicator results.

Variables	Definitions	Values	Cronbach’s Alpha	KMO
Cognition	You think GM crops are of better quality	1=Yes 0=No	0.763	0.737
	You think GM crops have higher yields			
	You think GM crops require less pesticide input			
	You think GM crops require less labor input			
	The relationship between eating GM food and human health	1=Harmful 2=Neutral 3=Beneficial		
	The relationship between feeding GM feed and livestock health			
	The relationship between growing GM crops and the balance of nature			
Risk perception	Do you think the climate conditions will get worse in the future	1=Yes 0=No	0.789	0.735
	Do you think pests will be more serious in the future?			
	Do you think soil conditions will deteriorate in the future?			
	Do you think the ecological environment will deteriorate in the future?			
	Do you think the maize market environment will deteriorate in the future?			
Value-oriented	When planting GM crops, I will prioritize my own interests.	1=Yes 0=No	0.757	0.661
	When planting GM crops, I don’t worry about fall armyworms damaging other people’s farmland.			
	When planting GM crops, I don’t consider the country’s strategic needs.			
	When planting GM crops, I don’t consider the impact on nature.			
Social trust	I believe in the GMO technology promoted by the government.	1=Yes 0=No	0.836	0.828
	If a relative suggests growing GM crops, I am willing to try.			
	If a neighbor suggests growing GM crops, I am willing to try.			
	If a village official suggests growing GM crops, I am willing to try.			
	If agricultural extension worker recommends planting GM crops, I am willing to try it.			
	If a seed company recommends growing GM crops, I am willing to try.			

Additionally, drawing on previous studies,^22,40,44^ this study also controls for variables such as household head’s age, household head’s education level, cultivated land area, training participation, and information sources. Training participation is measured by asking, “How many training sessions on genetically modified topics have you attended in the past year?” Information sources refer to the aggregated channels through which the household acquires information about genetically modified technologies.

### 3.3. Model Settings

Since the dependent variable is an ordered multi-classification variable, this research constructs an ordered Logit model to explore the impact of cognition, risk perception, value orientation and social trust on farmers’ adoption of genetically modified insect-resistant maize varieties:

(1) $$\mathrm{logit}[P(Y\leq i|X)]=\ln[\frac{P(Y\leq i|X)}{1-P(Y\leq i|X)}]=\beta_{0i}+\beta_{1}X_{1}+\beta_{2}X_{2}+\ldots +\beta_{m}X_{m}$$

Where Y represents farmers’ willingness to adopt insect-resistant GM maize varieties (Dependent Variable); $X_{i}$ denotes the explanatory variables, including knowledge, risk perception, value orientation, social trust, and other control variables. P(Y≤i|X) indicates the cumulative probability that Y falls into category *i* or lower given the independent variables X; $\ln\left(P/1-P\right)$ represents the log-odds; $\beta_{0i}$ is the model intercept term; and $\beta_{m}$ is the regression coefficient of the independent variables, indicating the change in farmers’ adoption willingness for each unit increase in X, which in turn affects the category probabilities.

From the above, we can see that the probability of Y taking 1 is:

(2) $$P_{1}=P(Y\leq 1|X)=\frac{1}{1+\exp\left[\left(-\beta_{01}+\beta_{1}X_{1}+\beta_{2}X_{2}+\ldots +\beta_{m}X_{m}\right)\right]}$$

Then, the probability of Y being 2 to 5 is:

(3) $$P_{2}=P(Y\leq 2|X)=\frac{1}{1+\exp\left[-\left(\beta_{02}+\beta_{1}X_{1}+\beta_{2}X_{2}+\ldots +\beta_{m}X_{m}\right)\right]}\;-\frac{1}{1+\exp\left[-\left(\beta_{01}+\beta_{1}X_{1}+\beta_{2}X_{2}+\ldots +\beta_{m}X_{m}\right)\right]}$$

(4) $$P_{3}=P(Y\leq 3|X)=\frac{1}{1+\exp\left[-\left(\beta_{03}+\beta_{1}X_{1}+\beta_{2}X_{2}+\ldots +\beta_{m}X_{m}\right)\right]}\;-\frac{1}{1+\exp\left[-\left(\beta_{02}+\beta_{1}X_{1}+\beta_{2}X_{2}+\ldots +\beta_{m}X_{m}\right)\right]}$$

(5) $$P_{4}=P(Y\leq 4|X=\frac{1}{1+\exp\left[-\left(\beta_{04}+\beta_{1}X_{1}+\beta_{2}X_{2}+\ldots +\beta_{m}X_{m}\right)\right]}\;-\frac{1}{1+\exp\left[-\left(\beta_{03}+\beta_{1}X_{1}+\beta_{2}X_{2}+\ldots +\beta_{m}X_{m}\right)\right]}$$

(6) $$P_{5}=P(Y\leq 5|X)=\frac{1}{1+\exp\left[-\left(\beta_{05}+\beta_{1}X_{1}+\beta_{2}X_{2}+\ldots +\beta_{m}X_{m}\right)\right]}\;-\frac{1}{1+\exp\left[-\left(\beta_{04}+\beta_{1}X_{1}+\beta_{2}X_{2}+\ldots +\beta_{m}X_{m}\right)\right]}$$

In addition, in order to improve the reliability of the research results, this study also uses binary dependent variables and binary logit models for robustness testing.^22^ Specifically, when the area of GM crops planted by farmers in 2023 is greater than 0, the value is assigned to 1, indicating that the farmers are willing to adopt GM insect-resistant maize varieties, and vice versa. The specific model is as follows:

(7) $$\mathrm{logit}\left(P\right)=\ln\left[\frac{P}{1-P}\right]=\beta_{0}+\beta_{1}X_{1}+\beta_{2}X_{2}+\ldots +\beta_{m}X_{m}$$

Among them, P represents the probability that farmers are willing to adopt genetically modified insect-resistant maize varieties; 1−P represents the probability that farmers are unwilling to adopt genetically modified insect-resistant maize varieties; the meanings of $\beta_{0}$, $\beta_{m}$ and $X_{i}$ are the same as in model (1).

### 3.4. Data Analysis

This paper draws on the scale development and validity testing research in technology adoption^84^ and employs Stata 17.0 to conduct exploratory factor analysis (EFA) on the variables related to farmer cognition, risk perception, value orientation, and social trust, calculating the KMO value (Table 2).^a^^a.^The Kaiser-Meyer-Olkin (KMO) values for farmers’ knowledge, risk perception, value orientation, and social trust are 0.737, 0.735, 0.661, and 0.828, respectively, indicating that these variables are suitable for exploratory factor analysis. Additionally, Stata 17.0 was used to conduct principal component analysis to extract common factors of variables, followed by validity tests^b^^b.^The Cronbach’s Alpha value for each variable exceeds 0.7, indicating high reliability of the scale. and multicollinearity tests.^c^^c.^The condition number using scaled variables is less than 30, indicating no multicollinearity issues among the explanatory variables.^87^ Furthermore, based on the results of the ordered Logit regression, marginal effects analysis (MEA) was employed to assess the degree of influence of each variable. Subgroup analyses were also conducted based on whether farmers participated in the 2022 GM pilot project (1 = Yes; 0 = No).

## 4. Results

### 4.1. Ordered Logit Model Regression

Table 3 presents the estimation results of the baseline ordered Logit model (Model 1) and the extended ordered Logit model (Model 2), examining the impact of farmers’ knowledge, risk perception, value orientation, and social trust on their willingness to adopt insect-resistant genetically modified maize varieties. The entire dataset was used to evaluate the influence of farmers’ knowledge and other factors on the adoption willingness of GM technology, with regression coefficients and Odds Ratio (OR) values reported for each explanatory variable.

**Table 3.** Regression results of ordered logit model (N = 426).

Variables	Model 1: Baseline model		Model 2: Extended Model	
	(1) Coefficient	(2) OR	(3) Coefficient	(4) OR
Cognition	0.716*** (0.165)	2.047*** (0.339)	0.696*** (0.166)	2.006*** (0.333)
Risk perception	0.018 (0.145)	1.018 (0.148)	−0.264 (0.195)	0.768 (0.150)
Value-oriented	0.257*** (0.133)	1.293** (0.172)	0.267** (0.133)	1.307** (0.174)
Social trust	0.484*** (0.126)	1.617*** (0.078)	1.349*** (0.416)	1.260*** (0.108)
Age of household head	0.015* (0.009)	1.015* (0.009)	0.016* (0.009)	1.017* (0.009)
Education level of household head	0.011 (0.022)	1.012 (0.022)	0.012 (0.022)	1.012 (0.022)
Area of cultivated land	−0.003 (0.006)	0.997 (0.006)	−0.003 (0.006)	0.997 (0.006)
Training status	0.209** (0.086)	1.232** (0.107)	0.191** (0.087)	1.211 (0.105)
Information source	0.112** (0.055)	1.119** (0.061)	0.124** (0.055)	1.132 (0.062)
Risk perception× Social trust	/	/	0.469 ** (0.213)	1.599** (0.340)

*p < .1; **p < .05; ***p < .01; The numbers in parentheses indicate standard errors.

Farmers’ cognition significantly enhances their willingness to adopt insect-resistant genetically modified maize. The results in columns (1) and (2) of Table 3 show that the regression coefficient for farmers’ cognition is positive and significant at the 1% level, indicating a significant positive relationship between cognition and the willingness to adopt GM technology, thus validating Hypothesis 1. Compared to other farmers, those with more positive views and deeper cognition of GM crops are likelier to adopt insect-resistant GM maize varieties. Additionally, an OR of 2.047 suggests that for each unit of increased cognition, the willingness to adopt increases by 2.05. This finding confirms that cognition is central to farmers’ adoption intentions.

Farmers’ risk perception influences their willingness to cultivate GM insect-resistant maize varieties only under specific conditions. As shown in column (1) of Table 3, the estimated coefficient of risk perception is statistically insignificant, indicating that risk perception alone does not significantly affect farmers’ willingness to adopt GM insect-resistant maize. The absence of a direct effect suggests that risk perception alone is not a significant barrier to adoption, partially challenging Hypothesis 2. However, the results in column (3) of Table 3 reveal that the interaction term risk perception × social trust has a positive and statistically significant coefficient at the 5% level (β = 0.47), suggesting that the effect of risk perception is context-dependent and becomes salient only when interacting with other factors. Specifically, farmers with higher trust in the government, acquaintances, and agricultural extension agencies show a more substantial positive influence of pest and disease risk perception on their willingness to adopt GM insect-resistant maize compared to those with lower social trust. Notably, a one-unit increase in risk perception leads to a 59.9% increase in adoption willingness, partially supporting Hypothesis 2. This moderating effect underscores the buffering role of social trust in promoting agricultural technologies.

Egoistically oriented farmers are more inclined to adopt GM maize. As indicated by the regression results in Table 3, when holding other control variables constant, the estimated coefficient for value orientation is positive and statistically significant at the 1% level (β = 0.26), suggesting that value orientation has a positive influence on farmers’ willingness to adopt GM insect-resistant maize, thereby supporting Hypothesis 3. Specifically, a one-unit increase in farmers’ prioritization of personal interests is associated with a 29.3% increase in their willingness to cultivate GM maize.

Social trust significantly promotes farmers’ willingness to adopt insect-resistant GM maize. As shown in column (1) of Table 3, social trust significantly impacts farmers’ willingness to adopt insect-resistant GM maize varieties, with a positive regression coefficient (β = 0.48). This indicates that enhancing farmers’ trust in society increases the likelihood of adopting GM crops, supporting Hypothesis 4. Furthermore, column (2) results demonstrate that a 1% increase in social trust level raises the probability of adopting GM crops by 61.7%. This finding underscores the critical role of building social trust in agricultural extension efforts to promote technology adoption.

Farmers’ socio-economic characteristics significantly influence their cultivation decisions toward GM maize. As shown in column (1) of Table 3, the estimated coefficient for household head age is positive and statistically significant (β = 0.02), indicating that older household heads are more likely to adopt GM insect-resistant maize. Furthermore, farmers’ participation in training is positively associated with their willingness to adopt; specifically, holding other explanatory variables constant, each additional training session increases the probability of adoption by 20.9%. The coefficient for information sources is also positive and significant at the 5% level, suggesting that more information channels related to GM crops increase the likelihood of adoption. In contrast, the household head’s education level and the cultivated land area show no statistically significant effect on adoption intentions.

### 4.2. Marginal Effect Analysis

Cognition factors significantly influence farmers’ willingness to adopt GM maize, followed by social trust and value orientation. According to the results in column (5) of Table 4, a one-unit increase in cognition significantly raises the probability of expressing a strong willingness to adopt GM crops by 11.0 percentage points (β = 0.11, *p* < .05), indicating that cognition is a key determinant of adoption decisions. Similarly, each one-unit increase in social trust significantly increases the probability of adoption by 7.4 percentage points (β = 0.074, *p* < .05), confirming the critical role of social capital in the diffusion of agricultural technologies. In addition, a one-unit increase in value orientation – favoring personal benefits – leads to a statistically significant increase of 3.9 percentage points in adoption probability (β = 0.04, *p* < .05), suggesting that egoistic value judgments strongly reinforce farmers’ adoption intentions. In contrast, risk perception does not statistically affect adoption willingness (*p* > .1), indicating its limited influence in this context.

**Table 4.** Marginal effects of the baseline model (Model 1).

Variables	(1)None Y=1	(2)Slightly Y=2	(3)Moderate Y=3	(4)Strong Y=4	(5)Extreme Y=5
Cognition	−0.051*** (0.014)	−0.088*** (0.020)	−0.018*** (0.005)	0.047*** (0.013)	0.110*** (0.025)
Risk perception	−0.001 (0.010)	−0.002 (0.018)	−0.000 (0.004)	0.001 (0.010)	0.003 (0.022)
Value-oriented	−0.018** (0.010)	−0.032** (0.016)	−0.006** (0.004)	0.017** (0.009)	0.039** (0.020)
Social trust	−0.035*** (0.010)	−0.059*** (0.015)	0.012*** (0.004)	0.032*** (0.009)	0.074*** (0.019)
Controls	YES	YES	YES	YES	YES
N	426				

*p < .1; **p < .05; ***p < .01; The numbers in parentheses indicate standard errors; Controls refers to other control variables such as the age of the household head.

Social trust can buffer the adverse effects of risk perception. As shown in Table 5, with the increase in the level of willingness to adopt GM maize (Y), the estimated coefficient for the interaction term risk perception × social trust shifts from significantly negative (β =  − 0.04, p < .05) to significantly positive (β = 0.07, *p* < .05), indicating an apparent heterogeneity in the moderating effect of social trust across different levels of adoption willingness. Specifically, among farmers with low willingness to adopt (Y ≤ 2), social trust amplifies the effect of risk perception, while among those with high willingness (Y ≥ 4), it plays a compensatory role, mitigating the perceived risks.

**Table 5.** Marginal effects of the extended model (Model 2).

Variables	(1)None Y=1	(2)Slightly Y=2	(3)Moderate Y=3	(4)Strong Y=4	(5)Extreme Y=5
Cognition	−0.050*** (0.138)	−0.085*** (0.020)	−0.017*** (0.005)	0.045*** (0.013)	0.106*** (0.025)
Risk perception	0.019 (0.014)	0.032 (0.024)	0.006 (0.005)	−0.017 (0.013)	−0.040 (0.030)
Value-oriented	−0.019* (0.010)	−0.032** (0.016)	−0.007* (0.003)	0.017** (0.009)	0.041** (0.020)
Social trust	−0.097*** (0.033)	−0.164*** (0.050)	0.033*** (0.012)	0.088*** (0.029)	0.205*** (0.064)
Risk perception× Social trust	−0.034** (0.016)	−0.057** (0.026)	−0.011** (0.006)	0.031** (0.014)	0.071** (0.032)
Controls	YES	YES	YES	YES	YES
N	426				

*p < .1; **p < .05; ***p < .01; The numbers in parentheses indicate standard errors; Controls refers to other control variables such as the age of the household head.

### 4.3. Heterogeneity Analysis

Experience with planting GM crops amplifies the impact of farmers’ cognition on the adoption of GM technology. As shown in columns (1) and (3) of Table 6, the estimated coefficients for farmers’ cognition are significantly positive at the 1% and 5% levels, indicating that farmers’ cognition positively influences their willingness to plant GM insect-resistant maize varieties, regardless of whether they participated in the 2022 GM pilot project. Specifically, the inter-group difference in coefficients is significant (*p* < .05), suggesting that the impact of cognition on planting willingness is stronger for farmers in the participation group (0.91 > 0.54) compared to those in the nonparticipation group. In other words, experience with planting GM crops strengthens the influence of cognitive levels on the intention to plant GM maize varieties.

**Table 6.** Group differences in the impact of farmers’ cognition on their willingness to plant GM crops.

Variables	Participation Group		Control Group		SUEST
	(1) Coefficient	(2) OR	(3) Coefficient	(4) OR	
Cognition	0.910***(0.263)	2.484***(0.654)	0.541**(0.218)	1.718**(0.375)	0.99[0.032]
Risk perception	−0.048(0.233)	0.953(0.222)	0.058(0.192)	1.059(0.203)	0.13[0.723]
Value-oriented	0.422**(0.187)	1.526**(0.285)	0.106(0.200)	1.112(0.222)	1.03[0.310]
Social trust	0.345*(0.200)	1.708*(0.142)	0.572***(0.167)	1.565***(0.094)	0.61[0.043]
Controls	YES	YES	YES	YES	/
N	196		230		/

The participant group refers to the sample farmers who participated in the 2022 GM pilot project; the control group refers to the sample farmers who did not participate in the 2022 GM pilot project; Controls refers to other control variables such as the age of the head of household; *p < .1; **p < .05; ***p < .01; The numbers in parentheses indicate standard errors.

Value orientation has a more significant impact on the planting willingness of farmers with GM crop planting experience. As shown in the results of column (1) in Table 6, the estimated coefficient for value orientation is significantly positive (β = 0.42), meaning that, when other variables are held constant, for each unit increase in self-oriented value orientation, the impact on farmers’ willingness to plant GM insect-resistant maize increases by 52.6%. However, as indicated by the estimated results in column (3), the coefficient for value orientation is not statistically significant, meaning that value orientation does not affect the planting willingness of farmers who did not participate in the 2022 GM pilot project.

The experience of planting genetically modified crops reduces the impact of social trust on farmers’ adoption of GM technology. As shown in columns (1) and (3) of Table 6, the estimated coefficients for social trust are significantly positive, indicating that social trust positively influences farmers’ willingness to plant GM insect-resistant maize varieties, regardless of whether they participated in the 2022 GM pilot project. Additionally, the difference in coefficients between the participation and nonparticipation groups is significant (*p* < .05), suggesting that social trust has a greater influence on the planting intention of farmers in the nonparticipation group (0.57) compared to those in the participation group (0.35). In other words, experience with planting GM crops reduces the role of social trust in influencing the intention to plant GM maize varieties.

Moreover, the model estimation results indicate that risk perception does not have a significant impact on the willingness to plant GM crops, regardless of whether farmers participated in the 2022 GM pilot project.

### 4.4. Robustness Test

Table 7 presents the results of the robustness check for the alternative dependent variable and model. The results show that farmers’ cognition positively influences the willingness to plant GM insect-resistant maize varieties at a 1% significance level. Specifically, when other explanatory variables remain constant, each one-unit increase in farmers’ cognition level corresponds to a 0.14-unit increase in the likelihood of adopting GM crops. The estimated coefficient for value orientation is positive at a 5% significance level (β = 0.31), indicating that farmers’ value orientation is positively correlated with their willingness to adopt GM technology. In other words, the stronger the self-directed value orientation, the higher the likelihood of adopting GM crops. The regression coefficient for social trust is positive, suggesting that social trust significantly positively influences farmers’ intention to plant GM insect-resistant maize. Specifically, for each unit increase in social trust, the likelihood of planting GM crops increases by 9.0%. The estimated coefficient for risk perception is not statistically significant. Additionally, the significance and direction of other control variables are consistent with those in the baseline regression model.

**Table 7.** Robustness test results.

	(1) Logit	(2) Marginal Effect
Cognition	0.618***(0.192)	0.136***(0.040)
Risk perception	−0.025(0.169)	−0.006(0.037)
Value-oriented	0.305**(0.151)	0.067**(0.033)
Social trust	0.406***(0.140)	0.090***(0.030)
Controls	YES	YES
N	426 230	

*p < .1; **p < .05; ***p < .01; The numbers in parentheses indicate standard errors; Controls represents other control variables such as the age of the household head.

In conclusion, the results from the binary Logit model estimation are consistent with those from the baseline regression model, confirming that the baseline model’s results are robust and further supporting the research hypotheses (H1–H4) in this paper.

## 5. Discussion

In the context of the severe threat posed by the fall armyworm to China’s maize industry, planting genetically modified insect-resistant maize varieties has become one of the key strategies for the green control of the pest. However, the differences in research on the cognition and acceptance of GM crops can have complex implications for national decision-making. The baseline regression model of this study confirms that farmers with more positive cognition toward GM crops are more likely to adopt GM insect-resistant maize varieties. This finding is consistent with the conclusions of previous studies by Ghasemi et al.,^47^ Rabbanee et al.,^48^ Ali et al. (2016), and Zhang.^32^ When farmers believe that planting GM crops can reduce production costs or increase food yield, they are more likely to choose GM varieties.^37,85^ In practice, due to the widespread infestation of fall armyworms in Southwest China,^79^ farmers with a higher level of cognition regarding the insect-resistant traits of GM crops tend to adopt such varieties to mitigate pest-related losses.

This study finds a significant positive correlation between farmers’ level of social trust and their willingness to adopt GM maize, which aligns closely with earlier findings by Zhou,^75^ Feng et al.,^76^ and Dai and Wang.^78^ A deeper examination of the underlying mechanism reveals that, in the context of agricultural production in Southwest China, GM technology – an emerging agricultural innovation – often encounters challenges related to limited farmer awareness and a lack of trust during the early stages of dissemination.^74^ Against this backdrop, farmers’ decisions regarding adopting GM crops are primarily shaped by the structure of social networks and the channels through which information is disseminated.^83^ Government agencies serve as key nodes in rural social networks as policymakers and promoters, family members as sources of emotional and experiential support, and agricultural extension agents as providers of technical guidance. These actors convey information about GM technology through routine interactions and training programs and influence farmers’ attitudes and acceptance through demonstration effects and word-of-mouth communication.

Social interactions based on social trust also play a significant role in adopting GM insect-resistant maize. Prior research has indicated that the adoption of GM technology among farmers often occurs through neighborly communication, observation, and imitation.^40^ This diffusion model, rooted in social networks, fundamentally depends on a foundation of interpersonal trust among farmers. Therefore, the greater the trust farmers place in key actors – such as government authorities, relatives, and agricultural extension agents – the more accessible the channels they receive information on GM technology. In turn, this enhances their understanding of the potential benefits and risks of the technology and increases the likelihood of adopting GM insect-resistant maize varieties.

Moreover, the effect of risk perception on planting preferences varies significantly among farmer groups with different levels of social trust. Among farmers with a higher degree of social trust, risk perception plays a positive moderating role in their adoption decisions. This finding is consistent with the results of Ghasemi et al.,^55^ Kim,^56^ and Angulo and Gil,^54^ all of which confirmed that risk perception can positively influence farmers’ attitudes toward GM technology, thereby enhancing their willingness to adopt. This is mainly because when such farmers develop a strong awareness of agricultural risks – such as fall armyworm infestations – they tend to view GM insect-resistant maize as an effective risk mitigation strategy, supported by their trust in government agricultural policies, technical guidance from extension agents, and successful planting experiences of relatives and peers. In contrast, farmers with lower levels of social trust, even when perceiving the threat of pests and diseases, often remain skeptical about the safety and reliability of GM technology due to a lack of trust in external actors. As a result, their perceived risk fails to translate into actual adoption behavior. Furthermore, some studies have confirmed that risk perception can directly reduce the acceptance of GM technology.^58,59,61^ This is primarily because some farmers are concerned that GM crop cultivation may damage the ecological environment^60^ and threaten intergenerational sustainability,^57^ thus diminishing their willingness to adopt.

This study also finds that farmers with egoistic value orientations are more inclined to adopt GM insect-resistant maize. This result aligns with our research expectations, suggesting that self-oriented values are a key factor influencing farmers’ adoption of GM technology.^70–72^ One possible explanation is rooted in the notion that all human behavior is inherently self-interested.^69^ However, this finding stands in contrast to the ethical decision-making perspective that emphasizes the public good,^86^ which argues that farmers who prioritize public interest and ecological ethics tend to reject GM crops, primarily due to their concerns for collective welfare and adherence to moral norms.

Finally, this study reveals significant group heterogeneity in the factors influencing farmers’ willingness to adopt GM crops. The results indicate that prior GM planting experience significantly strengthens the positive effect of cognition on technology adoption, which may be attributed to the knowledge dissemination and technical support provided as part of the 2022 government pilot program.^79^ The heterogeneity analysis further shows that egoistic value orientation has a more pronounced effect on adoption willingness among experienced farmers. This is primarily because, under the threat of severe maize yield losses caused by fall armyworms in Southwest China, the primary motivation for participating in the pilot program was to mitigate economic losses through pest-resistant GM technology. Moreover, as planting experience accumulates, cognition’s influence becomes increasingly prominent, while social trust’s role diminishes. This suggests a dynamic substitution effect between cognition and trust, wherein direct production experience gradually reduces farmers’ reliance on external information sources.

## 6. Conclusion

As one of the key provinces in Southwest China’s maize production belt, Yunnan serves as a critical frontline in combating the invasion of the fall armyworm, offering significant potential for the application of genetically modified technology. Based on household survey data collected in 2023 from Pu’er city in Yunnan Province, this study investigates the factors influencing farmers’ willingness to adopt GM insect-resistant maize in pilot areas. The results of the econometric model indicate that farmers’ cognition, risk perception, value orientation, and social trust are the primary drivers of GM crop adoption decisions. Heterogeneity analysis, conducted based on farmers’ participation in the pilot projects, reveals that GM crop planting experience moderates the influence of cognition and social trust on the adoption of GM technology, while value orientation plays a more pronounced role among farmers with prior GM crop planting experience.

Based on the findings above, the following recommendations are proposed: First, strengthen knowledge dissemination on GM technology to enhance farmers’ scientific literacy. Positive perceptions of GM insect-resistant maize can increase farmers’ willingness to adopt it, underscoring the importance of knowledge. Education and training are critical avenues for knowledge dissemination, and relevant educational resources and media platforms should be fully utilized to provide training on GM technology, increasing farmers’ understanding and improving their cognition of GM crops. Second, build institutional trust and facilitate effective multi-stakeholder communication mechanisms. Social forces should be mobilized to actively participate in building institutional trust. The government should encourage experienced farmers to adopt GM maize to alleviate concerns among other farmers and enhance trust in government efforts. Experts should address farmers’ questions using simple and accessible language, enabling them to trust expert systems and make informed production decisions based on expert advice. The media should ensure fair, accurate, and objective reporting, allowing farmers to independently make value judgments. Third, provide risk management training to farmers to foster accurate risk perception. Necessary risk management training should be provided to guide farmers in correctly perceiving risks, helping them make informed decisions and adopt GM technology more confidently.

This study, grounded in the context of the fall armyworm invasion, examines farmers’ willingness to adopt GM insect-resistant maize in Southwest China. It enriches the theoretical understanding of agricultural technology adoption by demonstrating the relevance of the threat perception – technological response behavioral mechanism to the promotion of GM crops. It also broadens the application of value orientation theory by highlighting the strengthening effect of egoistic motivation on technology adoption under crisis conditions. The findings provide empirical evidence to support governmental agencies’ development of differentiated promotion strategies for GM technology. However, several limitations remain. First, the study relies exclusively on data from Yunnan Province, which may not adequately represent other major maize-producing regions in China. The external validity of the findings should be further tested across different scales of farming operations (e.g., cooperatives, family farms), diverse agroecological zones (e.g., Northeast China, Huang-Huai-Hai region), and multiple years (longitudinal tracking). Second, although the study incorporates measurements of cognition, risk perception, social trust, and value orientation, the comprehensiveness and precision of certain indicators warrant further exploration and refinement in future research.

## Data Availability

If justified, readers can access the data via: huangyanfang01@caas.cn.
